# Rapid and Laboratory SARS-CoV-2 Antibody Testing in High-Risk Hospital Associated Cohorts of Unknown COVID-19 Exposure, a Validation and Epidemiological Study After the First Wave of the Pandemic

**DOI:** 10.3389/fmed.2021.642318

**Published:** 2021-08-26

**Authors:** Brendan O'Kelly, Ronan McLaughlin, Roseann O'Doherty, Hailey Carroll, Roisin Murray, Rachel Dilworth, Laura Corkery, Aoife G. Cotter, Tara McGinty, Eavan G. Muldoon, Walter Cullen, Gordana Avramovic, Gerard Sheehan, Denise Sadlier, Michaela Higgins, Peter O'Gorman, Peter Doran, Rosanna Inzitari, Sinead Holden, Yvonne O'Meara, Sean Ennis, John S. Lambert

**Affiliations:** ^1^Infectious Diseases Department, Mater Misericordiae University Hospital, Dublin, Ireland; ^2^Oncology Department, Mater Misericordiae University Hospital, Dublin, Ireland; ^3^Haematology Department, Mater Misericordiae University Hospital, Dublin, Ireland; ^4^Nephrology Department, Mater Misericordiae University Hospital, Dublin, Ireland; ^5^Centre for Experimental Pathogen Host Research, University College Dublin, Dublin, Ireland; ^6^School of Medicine, University College Dublin, Dublin, Ireland; ^7^Clinical Research Centre, University College Dublin, Dublin, Ireland

**Keywords:** COVID-19, SARS-CoV-2, rapid antibody test, seroprevalence SARS-CoV-2, high-risk hospital cohorts, first wave

## Abstract

**Objective:** We aimed to use SARS-CoV-2 antibody tests to assess the asymptomatic seroprevalence of individuals in high-risk hospital cohorts who's previous COVID-19 exposure is unknown; staff, and patients requiring haemodialysis or chemotherapy after the first wave.

**Methods:** In a single Center, study participants had five SARS-CoV-2 antibody tests done simultaneously; one rapid diagnostic test (RDT) (Superbio Colloidal Gold IgM/IgG), and four laboratory tests (Roche Elecsys® Anti-SARS-CoV-2 IgG [RE], Abbott Architect i2000SR IgG [AAr], Abbott Alinity IgG [AAl], and Abbott Architect IgM CMIA). To determine seroprevalence, only positive test results on laboratory assay were considered true positives.

**Results:** There were 157 participants, of whom 103 (65.6%) were female with a median age of 50 years (range 19–90). The IgG component of the RDT showed a high number of false positives (*n* = 18), was inferior to the laboratory assays (*p* < 0.001 RDT vs. AAl/AAr, *p* < 0.001 RDT vs. RE), and had reduced specificity (85.5% vs. AAl/AAr, 87.2% vs. RE). Sero-concordance was 97.5% between IgG laboratory assays (RE vs. AAl/AAr). Specificity of the IgM component of the RDT compared to Abbott IgM CMIA was 95.4%. Ten participants had positivity in at least one laboratory assay, seven (9.9%) of which were seen in HCWs. Two (4.1%) hematology/oncology (H/O) patients and a single (2.7%) haemodialysis (HD) were asymptomatically seropositive. Asymptomatic seroprevalence of HCWs compared to patients was not significant (*p* = 0.105).

**Conclusion:** HCWs (9.9%) had higher, although non-significant asymptomatic seroprevalence of SARS-CoV-2 antibodies compared to high-risk patients (H/O 4.1%, HD 2.7%). An IgM/IgG rapid diagnostic test was inferior to laboratory assays. Sero-concordance of 97.5% was found between IgG laboratory assays, RE vs. AAl/AAr.

## Introduction

Asymptomatic carriage of SARS-CoV-2 virus was identified early in the course of the pandemic and the potential infectivity of these patients has been speculated upon since that time. Early studies suggested asymptomatic COVID-19 may be highly transmissible (1). Quantification studies show this is not the case (2), but asymptomatic infection is still likely to be an important factor in the transmission dynamics of the virus with reported secondary infection between 5 and 18% (3–5).

Large seroprevalence studies of populations which included asymptomatic and symptomatic individuals during and after the first wave were done across the globe and showed low levels of seropositivity. Seroprevalence in Wuhan varied from 3.2 to 3.8% from 9th March to 10th April (6), 4.65% on April 10–11 2020 in Los Angeles County California (7), and 1.79% in Idaho in testing of 4,856 individuals over 1 week in April (8). Seropositivity was between 1.0 and 6.9% across 10 U.S. sites between March and April 2020 (9). The SEROCoV-POP study in Geneva, Switzerland of 2,766 individuals showed seroprevalence as high as 10.9% (10). The national seroprevalence rate in Ireland was estimated to be 1.7% in June/July 2020 at the same time of this study (11).

Serial hospital attendance is a necessary endeavor for some high-risk patients including those attending hematology and oncology (H/O) out-patient services and patients receiving haemodialysis (HD). These attendances may increase exposure to COVID-19 in these immunocompromised cohorts. In studies performed prior to the availability of vaccination for COVID-19, oncology patients were shown to have both higher seroprevalence of SARS-CoV-2 antibodies (12) and worse outcomes compared to the standard population (13, 14). Similarly, HD patients had worse outcomes than the standard population (15, 16), prevalence of infection in a HD unit was reported as high as 41.1% in one center, 40.5% of whom had no symptoms at the time of virus detection (17). Healthcare workers (HCWs) also represent a cohort with a higher incidence of COVID-19, occupational exposure to asymptomatic patients may be a factor in this (18).

The aim of this study was to assess the asymptomatic seroprevalence of SARS-CoV-2 infection in high-risk patient cohorts that have unavoidable hospital attendances using a rapid SARS-CoV-2 antibody test and laboratory serological assays. Additionally, the seroprevalence of HCWs was also investigated. A secondary aim was to determine the agreement of the rapid diagnostic test (RDT) with laboratory testing in both groups. Assessing the accuracy of antibody tests in real world studies is critical to determining their clinical utility (19).

## Methods

This study was designed as a single center 3-day prospective cohort study. The study was done in the Mater Misericordiae University Hospital (MMUH), Dublin, a 580-bed tertiary referral center which contains the National Isolation Unit (NIU) for Ireland. MMUH has treated over 450 in-patients with COVID-19 since its first case on the 3rd March 2020.

### Inclusion and Exclusion Criteria

Candidates were required to be patients or staff of the H/O directorate, be dialysis patients attending MMUH, be over 18 years of age and have capacity to consent to be included in the study. Individuals were excluded from the study if they had ever had a diagnosis of COVID-19 with confirmatory nasopharyngeal (N-P) polymerase chain reaction (PCR) testing, if they had typical symptoms of COVID-19 at the time of recruitment, or if they were currently receiving intravenous immunoglobulin (IVIG) as reports of reactive antibodies to SARS-CoV-2 in commercially available IVIG have been reported (20).

Although participants were asymptomatic for typical symptoms of COVID-19 at the time of the study, this cohort represents individuals whose prior exposure to COVID-19 since the onset of the first wave in March 2020 is unknown and unconfirmed, as routine antigen testing was not available at that time.

### Antibody Tests

Four SARS-CoV-2 commercially available antibody detection tools were used. A rapid antibody IgM/IgG colloidal gold test produced by Superbio, Jiangsu. This RDT is CE approved in Europe and pending FDA approval in the United States. Literature provided by the company report sensitivity of 95.3% and specificity 98.2% and consistency value between serum, plasma and whole blood at 100%. No cross reactivity was reported in samples with antibody positivity for influenza A/B, coronavirus (CoV), respiratory syncytial virus (RSV), *Haemophilus influenzae*, and anti-nuclear antibody (ANA). The comparative laboratory based IgG automated serological assays used were; Abbott Architect i2000SR (AAr) chemiluminescent microparticle immunoassay (CMIA) (Abbott Diagnostics, Chicago, USA) which has demonstrated 8.6% (<6 days) to 100% (>14 days) sensitivity, and specificity of 99.9% (8, 21), and Abbott Alinity (AAl) i SARS-CoV-2 IgG CMIA, negative percent agreement (NPA) of 99.63%, and a positive percent agreement (PPA) of 100% (in those 14 days post symptoms) (22), and Roche Elecsys® IgG (RE) electrochemiluminescence immunoassay analyser (ECLIA), sensitivity 100% and specificity 99.81% (23). The Abbott Architect i2000SR CMIA IgM was used as a standard to compare the IgM component of the IgM/IgG RDT, the laboratory assay demonstrates 99.56% specificity and 95% sensitivity in those tested 14 days post infection (24). All assays target the viral nucleocapsid. The assays were performed at the University College Dublin (UCD) Clinical Research Center (CRC) School of Medicine and Medical Science.

### Study Design

Consenting participants had a finger prick RDT and a serum sample taken at the same time for comparative laboratory serological assays. Day one of the study was conducted on the 24th June 2020 in the H/O directorate and included all patients on 31-single bed ward, all patients attending the H/O day-ward on that day and all associated staff members including staff nurses, nurse managers, research nurses, doctors, phlebotomists, healthcare assistants (HCAs), allied health professionals, and administrative staff. Days 2–3 of the study were conducted on the 5th−6th July 2020 in the 22 bed HD unit, where 72 patients encompassing the complete dialysis cohort of MMUH were considered for eligibility.

### Interpreting Test Results

The RDT test results were interpreted by two study team members together and were categorized as strongly positive, weakly positive, equivocal or negative, as follows: band intensity similar to the control band were deemed strongly positive, faint bands seen only in direct light were equivocal, while the range of band intensities between these two classifications were considered weakly positive. No quantifiable techniques apart from direct visualization were used to determine band intensity in order to replicate the qualitative visual assessment of real-world use. This scoring system was applied to bands at both the IgM and IgG positions of the RDT cassette. Equivocal bands were deemed positive when compiling RDT results, as SARS-CoV-2 cannot be entirely excluded in these cases and no guidance of an “exclusion threshold” of band intensity was offered with the literature provided by the manufacturers.

For the laboratory assays, manufacturer recommended indices of positivity were applied; cut-off index (COI) for AAl was ≥1.40, AAr ≥ 1.40 (both IgM and IgG), and RE ≥ 1.0. The IgG AAl and AAr are Abbott tests on different systems, and are the same test (AAl/AAr). In essence for IgG, three individual platforms were used the RDT, RE, AAl/AAr. A positive test on any laboratory assay was considered a true positive result.

Participants with IgM positive/equivocal test results on the RDT, had GeneXpert® N-P PCR testing performed at the time of antibody testing. Laboratory IgM testing using Abbott Architect CMIA was done on the 9th October 2020, once the test was validated and FDA approved.

### Managing Test Results

Results of the RDT were given to participants with the understanding that testing was done in the context of a research study. No decisions to isolate participants were made on the basis of the results of the rapid antibody test. Participants with an IgM positive band on the RDT who were subsequently found to be positive on the validated N-P PCR positive would then proceed through established COVID-19 pathways within the hospital. It was explained to patients during enrolment that if a positive RDT IgM is found, they may be precluded from treatment on the day of the study or offered admission to the hospital in their best interest, depending on the results of the subsequent N-P PCR test.

### Statistical Analysis

Data was compiled in Microsoft Excel® 2019. Descriptive analysis was done on collected data. McNemar's test was used to compare the differences in proportions of positive tests between the RDT and IgG serological assays. This was done separately for both RE and AAl/AAr. McNemar's test was also used to compare the differences in proportions for positive tests from the IgM component of the RDT to Abbott's IgM assay. The specificities of the IgM and IgG components of the RDT were calculated compared to the laboratory assays. Fisher's exact test was used to determine if seropositivity in HCW's compared to patient cohorts was significant. The statistical software used was SPSS V26.0.

The cumulative prevalence of COVID-19 was retrospectively calculated for each cohort (H/O patients, H/O staff, HD patients) by adding seropositive results of the study to the previously confirmed COVID-19 cases that were excluded and deemed not eligible for antibody testing.

### Ethics

Ethical approval was sought and approved by the local research ethics committee at MMUH. All participants provided written informed consent prior to testing.

## Results

**Figure 1:**
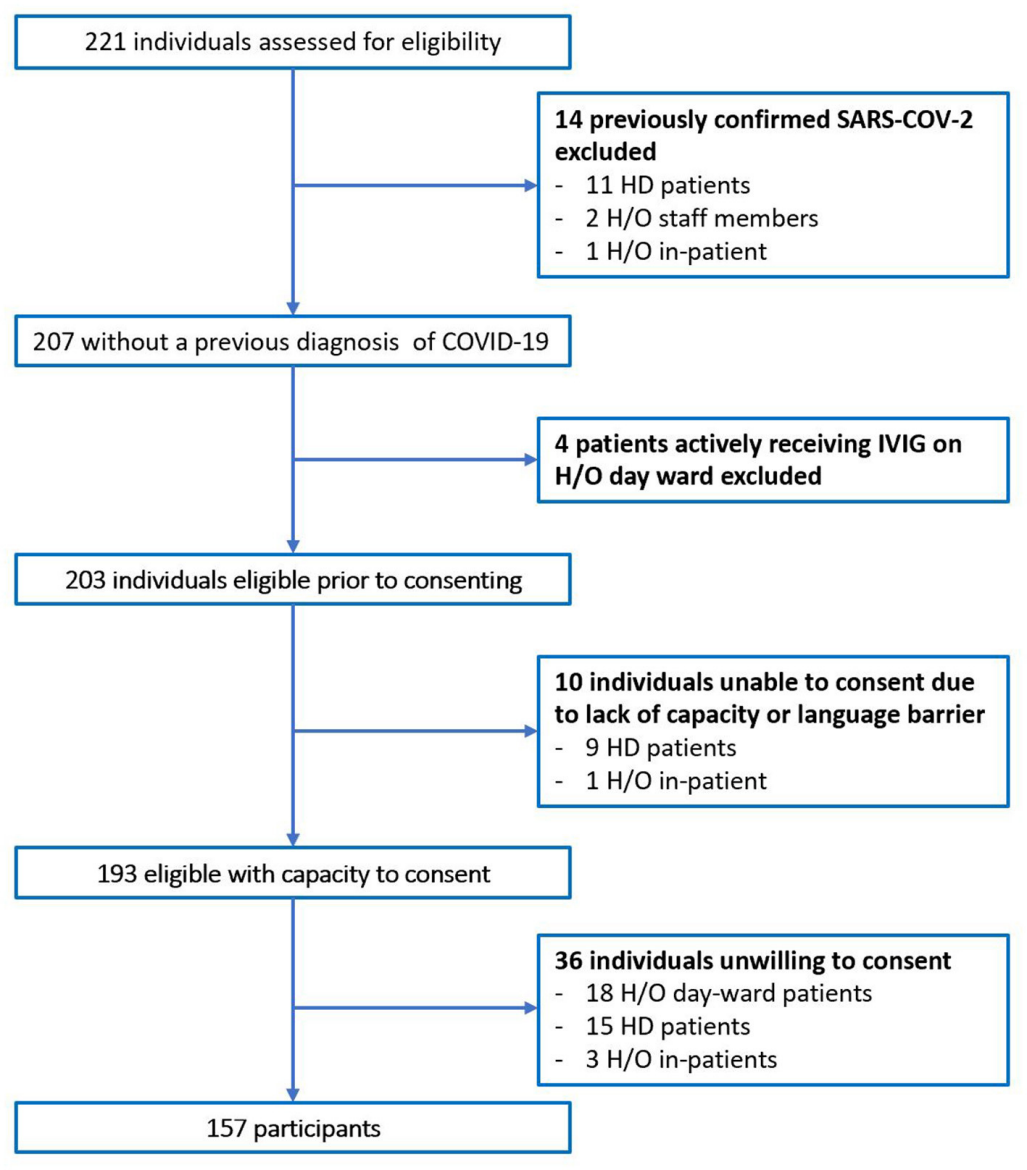
Flow diagram of excluded patients.

In total 221 individuals were assessed for eligibility. Of 64 individuals excluded, 14 had previously confirmed COVID-19 on N-P PCR swab, Figure 1. No participants with typical COVID-19 symptoms were identified during recruitment. The total number of participants included was 157. The majority were female (65.4%), median age was 50 years old (Range 19–90), and predominate ethnicity was Caucasian (87.3%). Participants included 71 (45.2%) H/O staff, 49 (31.2%) H/O patients, and 37 (23.6%) HD patients, Table 1. Diagnosis and treatment of H/O patients can be seen in the Supplementary Appendix 1.

**Table 1:** Demographics of participants tested.

**Total (*N* =157)**
Male	54 (35.4%)
Female	103 (65.6%)
Age-years (median)	50 (range 19–90)
**Ethnicity**
- Caucasian	137 (87.3%)
- Non-Caucasian	20 (12.7%)
H/O directorate patients total	49 (31.2%)
- In-patients	26 (16.6%)
- Day-ward patients	23 (14.6%)
- On chemotherapy	45 (92%)
H/O directorate staff total	71 (45.2%)
- Nursing staff	30 (42.3%)
- Medical staff	22 (31%)
- Other staff	19 (26.8%)
Dialysis patients	37 (23.6%)
- Charlson Comorbidity Index (CCI) median(range)	6 (range 2–11)

### Rapid Test IgG Results

**Figure 2:**
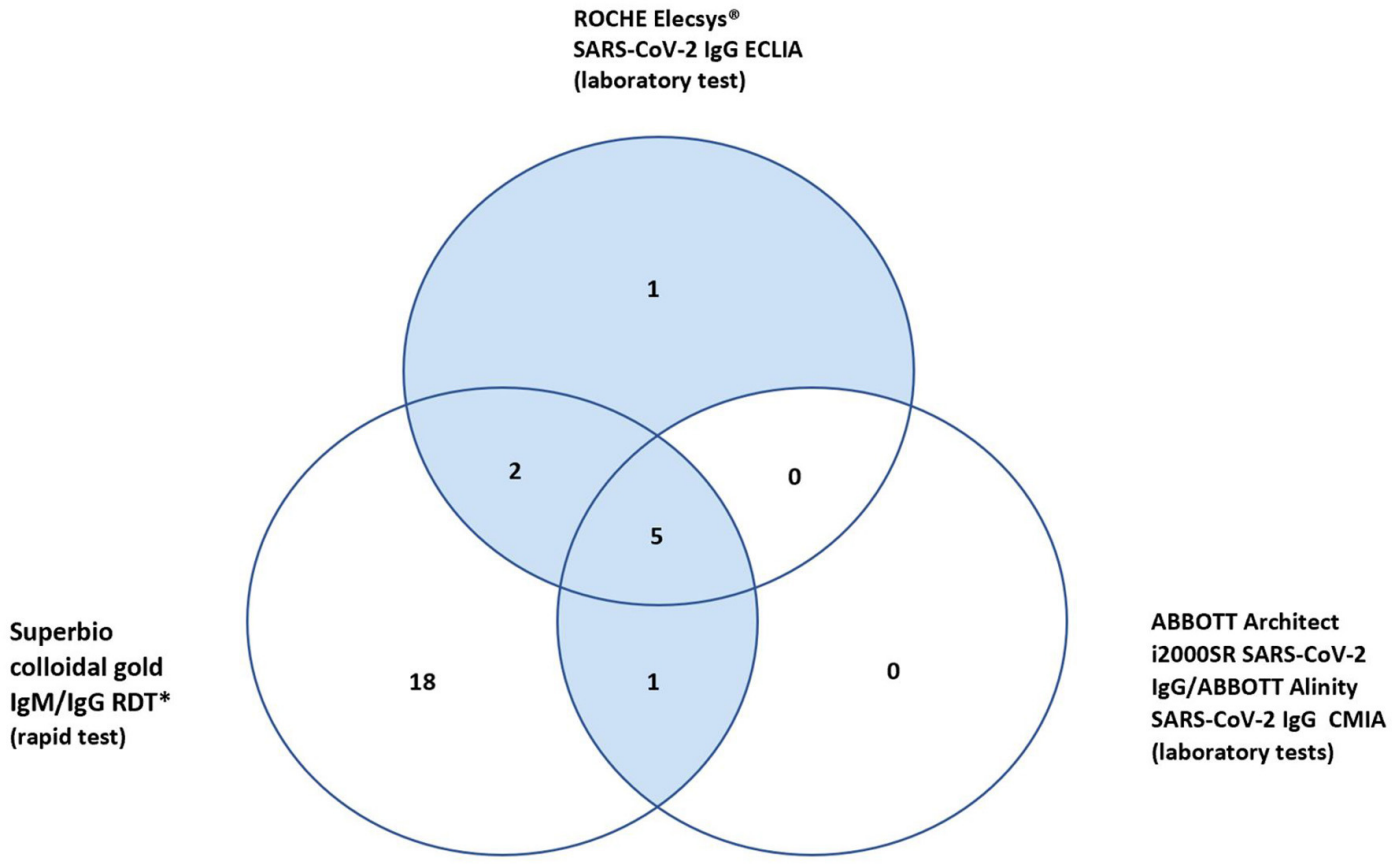
Venn diagram of positive IgG results.

In total 27 participants had a positive IgG on the RDT, 18 of whom were only positive on the RDT IgG and neither of the validated laboratory platforms (RE, AAl/AAr) (Figure 2). These 18 were deemed false positives. Using McNemar's test to compare the RDT to the laboratory tests it was found there was a statistically significant difference in both instances due to the high false positive rate (*n* = 157, RDT vs. RE *p* < 0.001 and RDT vs. AAl/AAr *p* < 0.001). A breakdown of positive IgG tests within staff and patient subgroups can be seen in Table 2.

**Table 2:** Positive results in each cohort and results of McNemar's test between RDT and laboratory assays.

		**Superbio Colloidal** **GOLD IgG**	**Superbio Colloidal** **GOLD IgM**	**Roche Elecsys®** **IgG ECLIA**	**Abbott Alinity/Architect i2000** **IgG CMIA**	**Abbott Architect** **IgM CMIA**	**McNemar's test**	**McNemar's test**	**McNemar's test**
							**IgG RE vs. RDT**	**IgG AAl/AAr vs. RDT**	**IgM Abbott vs. RDT**
							***p***	***p***	***p***
Total *n* (%)	157	27 (17.2%)	7 (4.5%)	8 (5.1%)	6 (3.8%)	4 (2.6%)	<0.001	<0.001	0.549
**Sub-group analysis**									
H/O patients	49	7 (14.2%)	4 (8.2%)	1 (2%)	0 (0%)	1 (2%)			
H/O staff	71	15 (21.1%)	2 (2.8%)	6 (8.5%)	5 (7%)	3 (4.2%)			
HD patients	37	5 (13.5%)	1 (2.7%)	1 (2.7%)	1 (2.7%)	0 (0%)			

### Rapid Test IgM Results

Seven participants had a positive IgM on the RDT and four participants were IgM positive on the Abbott CMIA platform. No participants were positive in both. All individuals who had positive RDTs at the time of the study had same-day N-P PCR swabs done on GeneXpert® platform and all were negative for SARS-CoV-2. McNemar's test indicate no significant difference between the samples (*p* = 0.549), the specificity of the IgM component of the RDT is 95.4%.

### Sero-Concordance of Laboratory Tests

In total ten (6.4%) of study participants had at least one positive validated laboratory test, Table 3. Three IgM/IgG positive, six IgG positive, and one IgM positive. Regarding the IgG laboratory-based assays; 100% sero-concordance was seen between AAl and AAr as expected. Five participants had a positive IgG in both RE and AAl/AAr. The overall sero-concordance between RE and AAl/AAr was 97.5%. Of the four discordant IgG participant results, three of these cases were positive in RE and negative in AAl/AAr, and one case was positive in AAl/AAr and negative in RE.

**Table 3:** Table of individuals with a positive test in at least one laboratory-based assay.

**Participant**	**Colloidal gold** IgG (RDT)^*^	**Roche Elecsys®**	**ABBOTT Architect**	**ABBOTT**	**Colloidal** **GOLD IgM**	**ABBOTT** **IgM** **CMIA**	**Participant**	**Sex**	**Previous N-P** **swab (±)**	**Previous symptom** **severity**
		**ECLIA IgG**	**i2000SR IgG**	**Alinity IgG**						
		**COI^α^ ≥ 1.0**	**COI ≥ 1.40**	**COI ≥ 1.40**		**COI ≥ 1.40**				
1	**Strong positive**	**120.8**	**4.52**	**5.68**	Negative	**2.69**	Staff nurse	F	No	asymptomatic
2	**Weak positive**	**3.58**	0.63	0.62	Negative	0.07	Doctor	M	Yes (–)	moderate: cough, sore throat, anosmia
3	**Strong positive**	**93.94**	**4.31**	**5.61**	Negative	**3.0**	Staff nurse	F	Yes (–)	moderate
4	**Strong positive**	**86.24**	**4.35**	**5.08**	Negative	**6.32**	Staff nurse	F	No	asymptomatic
5	**Strong positive**	0.064	**1.56**	**1.60**	Negative	0.04	Staff nurse	F	No	asymptomatic
6	**Weak positive**	**8.62**	0.16	0.14	Negative	0.03	Staff nurse	M	Yes (–)	moderate: general malaise 3 weeks
7	**Strong positive**	**43.01**	**2.26**	**2.30**	Negative	0.02	Staff nurse	F	Yes (–)	mild
8	**Strong positive**	**110.6**	**5.00**	**6.04**	Negative	0.21	Dialysis patient	M	No	asymptomatic
9	Negative	**1.6**	0.26	0.24	Negative	0.03	H/O in-patient	F	No	asymptomatic
10	**Weak Positive**	0.21	0.69	0.58	Negative	**1.46**	H/O out-patient	F	No	asymptomatic

* *The results of rapid antibody testing are included here but were not a contributing factor in deeming a participant positive. All results highlighted in bold were a positive result in that particular test*.

α *Cut-off Index*.

Of these ten seropositive findings on laboratory platforms seven were HCWs, conferring a 9.9% seroprevalence in asymptomatic staff. Two (4%) of H/O patients tested IgG positive and a single (2.7%) HD patient was seropositive. The difference between healthcare workers and patients was not found to be statistically significant, *p* = 0.103. Four of the ten seropositive participants had symptoms in the preceding weeks when asked retrospectively, all were negative on N-P PCR for SARS-CoV-2 when tested at the time of those symptoms.

### Cumulative Prevalence of COVID-19

Taking individuals with historical COVID-19 that were excluded prior to enrolment and adding them to the seropositive asymptomatic participants identified using laboratory serology tests, the cumulative prevalence of COVID-19 in these cohorts was calculated retrospectively; 6% (3/50) in H/O patients, 12.3% (9/73) of all staff members in the H/O directorate and 25% (12/48) of dialysis patients. The overall cumulative prevalence for all 157 participants was 14%.

## Discussion

In this study of 157 staff and patients associated with the hospital environment whose previous exposure to COVID-19 is unknown and who were asymptomatic at the time of the study, we found a SARS-CoV-2 seroprevalence of 6.4%. There was some variation in the seroprevalence of the individual cohorts although this did not reach significance (*p* = 0.103); 4% in H/O patient cohort, 2.7% HD cohort an 9.9% in staff members. When examining frontline workers with most patient contact i.e., nursing staff/medical staff in this study, we find an asymptomatic seroprevalence of 13.5% (7/52). Prevalence studies of COVID-19 in HCWs during and after the first wave have been done with variable findings. One study using both RT-PCR and antibody testing (includes asymptomatic and symptomatic staff at the time of the study) against the Spike protein (Euroimmun SARS-CoV-2 IgG) have found overall infection rates of 12.6%, although being a nurse/physician was not a risk factor for this (25). A study in a specialist infectious diseases directorate in Italy, found prevalence (RT-PCR plus serology, MAGLUMI 2019-nCoV IgM/IgG, spike and nucleocapsid) in asymptomatic staff as low as 3.4%, three of the four positive participants were either a nurse or physician (26). Another study of 249 HCWs in a single center in Kentucky reported 7.6% seroprevalence using serological testing targeting the SARS-CoV-2 spike protein in symptomatic and asymptomatic staff. Of the 19 positives, 11 (68.4%) were a physician or a nurse (27). A study of staff in a H/O directorate in Milan in April 2020 showed 6.9% (7/101) seropositivity amongst doctors and 11.3% (15/133) amongst nurses/paramedics/other staff members using PRISMA IgM/IgG targeting the SARS-CoV-2 nucleocapsid in asymptomatic, pauci-symptomatic, and symptomatic patients (28). Based on these limited studies there does appear to be a trend toward higher seroprevalence in staff members with the most exposure to patients i.e., physicians/nurses.

Regarding the types of antibody tests used in seroprevalence studies, some studies examine antibodies to either the viral nucleocapsid, Spike protein or both. In this study the antibody tests target the nucleocapsid alone. Anti-nucleocapsid serological testing has been found to be highly sensitive and specific; Muench et al. show that in a sample size of 10,453 patients at ≥14 days post PCR positivity, RE assay shows sensitivity of 99.5% and specificity of 99.8% (29). Large studies have adopted these tests for assessing seroprevalence. The PRECISE study examining antibodies in symptomatic and asymptomatic HCWs across two sites in Ireland; St James's Hospital and University Hospital Galway have used both RE and AAr in 5,787 staff members and found seroprevalence of 15% and 4.1%, respectively (30). The SCOPI study was also a national seroprevalence study in Ireland that used AAr for symptomatic and asymptomatic participants. Of 1,733 participants aged 12–69 an overall seroprevalence of 1.7% was found (11). It could be argued that in our study and the studies outlined above where antibodies against the nucleocapsid alone were used to identify seropositive patients there may be participants that have acquired COVID-19 and mounted antibodies to the Spike protein alone, therefore their antibody status would go unrecognized using anti-nucleocapsid assays. Conversely, very high agreement has been shown between antibody tests that target either the nucleocapsid or Spike protein; Prince et al. compare AAr with three assays targeting the Spike protein (DiaSorin Liaison, Ortho Vitros, and Euroimmun) and show a consensus negative interpretation from 96.7 to 100% and a consensus positive interpretation from 94.3 to 100% (31). In essence this effect may be small and using anti-nucleocapsid assays for seroprevalence studies is likely valid.

The RDT performed poorly due to its high false positive rate compared to RE and AAl/AAr. The RDT IgG component showed a lack of specificity ranging between 85.5 and 87.2% depending on which validated laboratory test it was compared to Table 4. Similar findings were also found in an FDA report, describing a specificity of 85% for the Superbio Colloidal Gold RDT (32). One factor attributing to this may be misinterpretation of the RDT results as a spectrum of band intensities were found. Although an association of stronger bands with true positives (*n* = 7 of 10) was found, this does not necessarily aid the user in real-world settings, where bands of any intensity cannot fully exclude presence of SARS-CoV-2 antibodies.

**Table 4:** Performance of the rapid test vs. laboratory tests.

	***N* = 157**	**True positive**	**False positive**	**True negative**	**False negative**	**Specificity**
Superbio Colloidal GOLD IgM	(vs. Abbott IgM)	0	7	150	4	95.4%
Superbio Colloidal GOLD	(vs. RE)	7	19	138	1	87.2%
IgG	(vs. AAl/AAr)	6	20	137	0	85.5%

The RDT IgM component showed more specificity (95.4%) when compared to the Abbott IgM test. Although none of the four laboratory IgM positives were positive on the RDT, inferring there may be a lack of sensitivity with the test.

There was good agreement between laboratory serology tests (RE, AAl/AAr) for SARS-CoV-2 IgG (97.5%). Harley and Gunsolus also show 98.7% agreement in a cohort of 667 (*n* = 103 COVID-19 confirmed, *n* = 564 pre COVID-19 samples) comparing RE and AAl (33). With only 10 seropositive results in our study, it is not sufficiently powered to determine the sensitivity of the serology tests.

In participants deemed positive without full consensus across laboratory tests (participants 2,5,6,9 Table 3), the results of negative tests in this group are below COIs for positivity (RE ≥ 1.0, AAl/AAr ≥ 1.40) but have values higher than truly negative individuals with no positive results. False positive results, cross-reactivity, and waning antibody levels may be explanations for this lack of consensus. A Cochrane review of 38 studies of antibody tests found false positive results in just 2% of cases, some variability was found depending on prevalence of COVID-19 within populations (34). Regarding waning antibody levels, a study examining levels of 34 mildly symptomatic individuals found an exponential decay of antibodies levels greater than that seen in SARS-CoV-1, with a half-life of 73 days over the study period (35). The first case of community acquired COVID-19 reported in the Republic of Ireland was on February 29th 2020 (36), by the date of enrolment for this study on 24th of June, antibody levels hypothetically could have fallen below the threshold of positivity for commercially available tests for participants infected early during the first wave. If this is the case, there may be under-reporting of truly positive participants in this study or any seroprevalence study.

There may also be some variability in the mounting of antibody responses between symptomatic and asymptomatic patients. In the case of The Diamond Princess cruise ship, of 215 individuals that were asymptomatic and initially N-P PCR negative, nine individuals subsequently swabbed positive and all nine went on to develop antibodies by day 8 (37). Another study did not find any association between IgG plateau levels and clinical severity of the disease (38). Conversely, it has been shown mild disease may be associated with reduced antibody response compared to severe disease (39). One study comparing six symptomatic with eight asymptomatic/mild infections found all six symptomatic individuals mounted IgG response, four of whom also mounted IgM. No asymptomatic/mildly symptomatic patients developed IgM and five of eight developed IgG antibodies (40). Interestingly it appears that even if antibody levels have fallen below the threshold of positivity there is some neutralization ability at least up to 6 months as was seen in a large study of 12,666 HCWs in the UK using that presence of anti-Spike IgG and/or anti- nucleocapsid IgG where very low levels of re-infection were found (41).

Overall, the cumulative prevalence (seroprevalence of asymptomatic study participants + PCR positive individuals excluded, Figure 1) of COVID-19 for this cohort of individuals with high-risk exposure was 14% (24/172). This value is vastly higher than the seroprevalence of the “background population” in Ireland at the time of 1.7% elucidated by the SCOPI study and suggests high risk hospital associated cohorts may be at increased risk of acquiring COVID-19 by a number of fold due to their needs to frequently engage with the hospital environment (11).

Although low seroprevalence levels were seen in both patient groups (H/O 4%, HD 2.7%) the cumulative prevalence of COVID-19 in these patient groups was calculated to be 6 and 25%, respectively. This does appear to be quite a difference for patients attending the same hospital. The COVID-19 management strategies were similar for both directorates. The single biggest difference in care was the transposition of the H/O day-ward to a repurposed nurse training center adjacent to the hospital; a step not feasible for the HD unit. Another likely important factor in the higher prevalence of COVID-19 in the HD group is the much higher frequency of visits HD patients require, i.e., three times per week. Also 92% (45/49) of H/O patients were receiving some form of chemotherapy, it is unclear to what extent immunosuppressants impact antibody levels in COVID-19 infection.

This study has a number of weaknesses. With a sample size of 157 from a single center, the study is not significantly powered to determine sensitivity of the serology tests used. Also, only assays targeting the nucleocapsid were used, there may have been participants who had acquired COVID-19 and had antibodies to the Spike protein alone that were not identified in this study. The patient cohorts in this study were immunocompromised and may not have mounted detectable antibody responses with the assays used and would therefore be false negative results. Testing was also done at a single time-point, taking serial serological samples through time would give a better understanding of how antibody levels change over time and a better understanding of the transmission dynamics of SARS-CoV-2 infection in hospitals. Enrolling participants from a uniform population i.e., sampling of asymptomatic staff members only, or high-risk H/O or HD patients across multiple sites would have provided more uniformity and robustness to the study. The results of this study are specific to the high-risk cohorts sampled (H/O patients/staff and HD patients) are not generalisable to all HCWs. We also highlight that although participants were asymptomatic at the time of the study the prior exposure to COVID-19 is unknown. We assume that patients and staff with typical symptoms would have been identified through screening pathways already in place within the H/O and HD directorates and would have had PCR testing. Those individuals identified as having a positive test would then be excluded as part of the exclusion criteria of this study.

## Conclusion

In conclusion, asymptomatic seroprevalence in high-risk hospital associated cohorts where previous COVID-19 exposure was unknown was 6.4% (*n* = 10 participants). Highest seroprevalence was in HCWs (9.9%). There was strong agreement in laboratory IgG antibody testing for SARS-CoV-2 with 97.5% sero-concordance between RE and AAl/AAr. The RDT had a high number of false positives, *n* = 18 (11.5%), and their clinical use cannot be supported by this particular study.

## Data Availability Statement

The raw data supporting the conclusions of this article will be made available by the authors, without undue reservation.

## Ethics Statement

The studies involving human participants were reviewed and approved by Mater Misericordiae University Hospital local research ethics committee. The patients/participants provided their written informed consent to participate in this study.

## Author Contributions

BO'K, RMc, RO'D, AC, TM, GA, WC, PO'G, PD, and JL: study design. BO'K, RMc, RO'D, HC, RMu, RD, LC, PO'G, RI, and PD: execution of study. BO'K, SH, and JL: statistical analysis. BO'K, RMc, RO'D, AC, TM, EM, WC, DS, MH, PO'G, PD, RI, SH, YO'M, SE, and JL: writing the paper. All authors contributed to the article and approved the submitted version.

## Conflict of Interest

The authors declare that the research was conducted in the absence of any commercial or financial relationships that could be construed as a potential conflict of interest.

## Publisher's Note

All claims expressed in this article are solely those of the authors and do not necessarily represent those of their affiliated organizations, or those of the publisher, the editors and the reviewers. Any product that may be evaluated in this article, or claim that may be made by its manufacturer, is not guaranteed or endorsed by the publisher.
