# Supplementary material for: Rapid and Laboratory SARS-CoV-2 Antibody Testing in High-Risk Hospital Associated Cohorts of Unknown COVID-19 Exposure, a Validation and Epidemiological Study After the First Wave of the Pandemic
Source: Front Med (Lausanne). 2021 Aug 26;8:642318. doi: 10.3389/fmed.2021.642318 (PMC8427142; doi:10.3389/fmed.2021.642318)
Supplement: Supplementary file 1 [file Data_Sheet_1.pdf]

Supplementary Appendix

S1 Malignancy subtype in in-patients and day ward patients

	n=
**In-patient diagnoses***	
DLBCL	7
MM	2
AML	3
ALL	1
breast ca	3
Lymphoma	5
HL	1
cervical ca	1
renal cell carcinoma	1
duodenal ca	1
AIHA	1
MDS	1
mucinous adenocarcinoma	1
Aplastic anaemia	1
**Day-ward patient diagnoses**	
Breast Ca	8
Ovarian Ca	4
Colon Ca	5
NSCLCa	1
MDS	2
Appendiceal Ca	1
Aplastic Anaemia	1
NHL	1
In-patients chemotherapy	24/26
Out-patients chemotherapy	21/23
Out-patients transfusion	2/23

*In-patients may have more than one diagnosis. DLBCL – diffuse large B cell lymphoma, MM – multiple myeloma, AML – acute myeloid leukaemia, ALL – acute lymphoblastic leukaemia, ca – cancer, HL – hodgkin's lymphoma, AIHA – autoimmune haemolytic anaemia, MDS- myelodysplastic syndrome, NSCLCa – non-small cell lung cancer, NHL – non-hodgkin's lymphoma
